# Food System Transformation: Integrating a Political–Economy and Social–Ecological Approach to Regime Shifts

**DOI:** 10.3390/ijerph17041313

**Published:** 2020-02-18

**Authors:** Laura M. Pereira, Scott Drimie, Kristi Maciejewski, Patrick Bon Tonissen, Reinette (Oonsie) Biggs

**Affiliations:** 1 Centre for Complex Systems in Transition, Stellenbosch University, Stellenbosch 7600, South Africa; scottdrimie@mweb.co.za (S.D.); krismacski@gmail.com (K.M.); oonsie@sun.ac.za (R.O.B.); 2 Centre for Food Policy, City University of London, London EC1V 0HB, UK; 3 Stockholm Resilience Centre, Stockholm University, 106 91 Stockholm, Sweden; 4 Copernicus Institute of Sustainable Development, Utrecht University, 3584 CB Utrecht, The Netherlands; 5 Center for European Integration Research, University of Bonn, 53113 Bonn, Germany; patrick.tonissen@ipbes.net

**Keywords:** food systems, global food regimes, innovation, political–economy, social–ecological systems, transformation, regime shifts, resilience

## Abstract

Sustainably achieving the goal of global food security is one of the greatest challenges of the 21st century. The current food system is failing to meet the needs of people, and at the same time, is having far-reaching impacts on the environment and undermining human well-being in other important ways. It is increasingly apparent that a deep transformation in the way we produce and consume food is needed in order to ensure a more just and sustainable future. This paper uses the concept of regime shifts to understand key drivers and innovations underlying past disruptions in the food system and to explore how they may help us think about desirable future changes and how we might leverage them. We combine two perspectives on regime shifts—one derived from natural sciences and the other from social sciences—to propose an interpretation of food regimes that draws on innovation theory. We use this conceptualization to discuss three examples of innovations that we argue helped enable critical regime shifts in the global food system in the past: the Haber-Bosch process of nitrogen fixation, the rise of the supermarket, and the call for more transparency in the food system to reconnect consumers with their food. This paper concludes with an exploration of why this combination of conceptual understandings is important across the Global North/ Global South divide, and proposes a new sustainability regime where transformative change is spearheaded by a variety of social–ecological innovations.

## 1. Introduction: The Need for Transformation of the Global Food System

Achieving the goal of global food security, together with environmental sustainability and social and economic justice, is one of the greatest challenges of the twenty-first century. The current food system is failing to meet the needs of people, and simultaneously harming the environment and undermining human well-being [1]. With a current world population of more than 7 billion people, expected to reach 9 billion by 2050, global demand for food is undeniably increasing [2]. Food price spikes since 2008 have dramatically affected the affordability of food for much of the world’s poor, with hunger on the rise again, largely due to the proliferation of violent conflict, climate-related shocks, and economic downturns [3,4]. As the world moves towards achieving the Sustainable Development Goals by 2030, goals 2 (*end hunger),* 3 (*improve health)*, 8 (*decent work and economic growth*), 12 (*responsible consumption and production*), 13 (*climate action*), 14 (*life below water*), and 15 (*life on land*), are all deeply interlinked with the global food system [1].

Added to the challenge is a burgeoning crisis regarding how we utilize food. The food system is failing to meet the needs of the 820 million undernourished people, 2 billion with micronutrient deficiencies, and more than 600 million diagnosed with obesity [5]. An ailing food system is exacerbating problems in the health system with severe health implications not only arising from under-nutrition and micro-nutrient deficiencies, but with obesity and overweight being linked to 44% of the diabetes burden, 23% of the ischemic heart disease burden, and 7–41% of certain cancer burdens [6,7]. Compounding these trends is the fact that with increasing affluence, diets are shifting dramatically towards more sugar, animal, and fat products, to the exclusion of traditional—and often more sustainable—diets [8,9].

The global food system, especially food production, is a major driver of global environmental change, and has driven massive transformations of terrestrial and marine ecosystems [10]. Human use currently directly affects more than 70% of global ice-free land, and estimates show that up to one third of terrestrial net primary productivity is being used for food, feed, fiber, timber, and energy [11]. A rapidly expanding aquaculture sector is occupying more terrestrial, coastal, and offshore space [12], and projections show that without comprehensive fisheries reforms, over 80% of global fish stocks will be overfished and below their critical biomass by 2050 [13]. Industrialized agriculture is highly reliant on external inputs, contributes to chemical pollution through the use of pesticides and herbicides, changes nitrogen and phosphorous cycles through the addition of synthetic fertilizers, and impacts freshwater stocks through irrigation [14,15]. It is also energy intensive, emitting almost one-third of all greenhouse gases, including methane, thereby contributing to climate change [16].

It is therefore increasingly apparent that a deep structural transformation is necessary in the way we produce and consume food to ensure a more just and sustainable future. The nature of the sustainability challenge means that previously dominant ways of doing things and understanding the world need to be reconsidered in order to make way for knowledge systems that can deal with accelerating change, increasing complexity, contested perspectives, and inevitable uncertainty [17]. This paper uses the concept of regime shifts to understand key drivers and innovations underlying past structural shifts in the food system, and explores how this understanding may help us think about leveraging change to enable a better food system in the future. The paper combines two perspectives on regime shifts: the political–economy perspective derived from the social sciences, and the social–ecological systems perspective derived from the natural sciences. Through an interdisciplinary understanding of food regimes, the paper suggests how connecting different disciplinary understandings of food system change can help to reimagine alternative innovations towards sustainability.

The paper begins with an introduction to regime shifts from the political–economy and social–ecological systems literatures, and then develops an interpretation of food regimes in three case studies drawing from innovation theory. It concludes with an example from a Global South context that highlights how this interdisciplinary conceptualization of regime shifts can be important in highlighting transformative pathways towards a more sustainable future.

## 2. Two Conceptualizations of Food Regime Shifts

There are a number of frameworks and literature that reference the term “regime” as the dominant way in which processes operate within a system, which is typically associated with distinct system structures. In this paper, we focus on two uses of the regime concept that come from distinct disciplinary backgrounds: (1) the concept of global food regimes introduced by Friedmann and McMichael [18] that emerges from a political–economy perspective, which identifies a regime as a stable period of capital accumulation that is associated with particular configuration of geopolitical power; and (2) a more ecological understanding of regimes that has its foundations in social–ecological systems or resilience thinking, where a regime refers to the combination of factors that constrain the way an (eco) system is structured and functions [19]. By combining the social–ecological and political–economy concepts of regime shifts, we aim to understand past changes in the food system and how future changes might be nudged onto more desirable trajectories.

### 2.1. A Political–Economy Framing of Food Regimes

In 1989, Friedmann and McMichael published a seminal paper that identified two global food regimes, i.e., the diasporic-colonial food regime of 1870–1914, and the mercantile-industrial food regime of 1947–1973 [18]. The food regime analysis highlighted the important role of food in the global political–economy and provided a structured approach to understanding the role of capital accumulation in agriculture, expressed in the patterns of food circulating in the world economy [20]. Their work also demonstrated a clear shift from one food regime to another during the twentieth century, and linked international modes of food production and consumption to specific periods of capital accumulation.

The diasporic-colonial food regime of 1870–1914 was defined predominantly by food imports to Europe from the colonies; basic grains and livestock from the colonial territories, most notably Australia, Canada, and the United States of America, and tropical imports from the rest of the occupied colonies [20]. By outsourcing its food production, Britain in particular was able to provide its industrial class with cheap food, thereby delineating ‘development’ in the twentieth century as a dynamic between national agricultural and industrial sectors [20]. In contrast, the mercantile-industrial food regime of 1947–1973 rerouted food from the USA “to its informal empire of postcolonial states on strategic perimeters of the Cold War” [20]: p.141. This shift to a new food regime occurred after the two World Wars, which heralded massive changes in global geo-political power. Framed as a development project that consisted of a suite of interventions such as food aid, Green Revolution technologies, and the extension of markets into the countryside, this regime “universalized a national model of economics as central to the state system following decolonization, whilst simultaneously creating a new international division of labor in agriculture that was centered on transnational commodity complexes” (Raynolds et al. 1993 in [21]: p.141).

In 2005, Friedmann suggested that a third corporate-environmental regime had emerged [22] as the dominance of transnational retailers and agro-food companies—or ‘Big Food’—had created a global impact. The premise is that globally powerful food retailers and agro-food companies selectively appropriated demands from environmental and social movements for their own purposes. Friedmann grounds her argument in the restructuring between regimes rather than on the periods of stability, thereby arguing that this food regime emerged out of the contestations between social movements and powerful institutions, which resulted in a new institutional frame [22]. The rise of the third regime is arguably a response to the critique by environmental and social movements of industrial agriculture that began during the pinnacle of the mercantile-industrialist regime in the 1960s. Such critiques put forward alternatives such as ‘organic’ and ‘local’, which led to experimentation with agro-ecological practices in the Global North. As these debates unfolded, in the Global South, the Green Revolution and industrial agriculture were transforming agro-ecosystems to increase the yields of staple crops whilst marginalizing rural communities and eroding agro-biodiversity and indigenous knowledge [22]. The rise of this new regime has arguably pivoted on the capture of these environmental critiques and movements by the more powerful corporations to reconfigure the mercantilist regime away from the influence of nation state actors to sit squarely in the hands of the private sector [23]. The appropriation by transnational corporations of the language of social movements has largely captured the rhetoric and façade of sustainability while broadly failing to meet the health and environmental needs of the communities being serviced. Ongoing research on how private food system actors are configuring food environments to the detriment of the health of many communities reinforces this dilemma and, at the same time, shifts the problem space from agricultural production to the dimension of access and consumption [24,25,26].

Concerns about food consumption patterns within food regimes have been highlighted by authors such as Dixon [27], who traced the history of the ‘imperial calorie’ through the ‘protective’ vitamin and more recently the ‘empty calorie,’ linking these to contemporary concerns around the ‘nutrition transition’ and rising levels of obesity [28]. The use of the term food deserts—places where it is almost impossible for people to be able to access affordable, healthy food—has sparked contestations in the food system that go beyond food production and engage more substantially with issues of accessibility, nutritional content and cultural appropriateness [29,30,31,32,33,34,35]. Such analyses open up questions of how individuals value and interact with their food environment and how understanding these everyday practices can offer non-paternalistic solutions to the crisis of the food system that gives agency to communities to instigate change, rather than relying either on the state or the private sector to save them [34]. However, the focus on consumption remains broadly within the domain of health. Although implicit in many political–economy analyses of food regimes, the environmental implications remain largely side-lined. One exception is Campbell [36], who has used resilience theory to include ecological dynamics into the political–economy analysis of food regimes. His work provides a segue from the political–economy description of food regimes to a social–ecological analysis.

### 2.2. Social–Ecological Regime Shifts in the Food System

Campbell’s use of resilience theory as a way to incorporate ecological feedback into the political–economy perspective of food regimes illustrates that there are advantages to bringing these perspectives closer together because they emphasize different aspects of what is essentially a problem of an inequitable and unsustainable food system [36]. There is an increasing consensus that the food system can be conceptualized as a complex social–ecological system: it consists of cross-scale, multilevel interactions between humans and natural systems, has emergent properties and tipping points, and exhibits nonlinear behavior arising from the interactions and feedbacks in the system [37,38]. Social–ecological regime shifts are defined as long-lasting shifts or changes in the structure and function of social–ecological systems that occur when a tipping point is crossed and a different set of system feedbacks and processes become dominant, restructuring the way the system operates. Such shifts can be precipitated by large shocks as well as slower changes that disrupt or weaken the previously dominant processes and feedbacks [19]. Examples of shocks that can trigger social–ecological regime shifts include climatic fluctuations, large storms, fires, disease, or the outbreak of conflict. Slow changes that may push a social–ecological system towards a tipping point include the gradual consolidation of small farms into large corporate entities, which changes the distribution of benefits and power in the food system, as well as the diversity of food produced.

**Figure 1:**
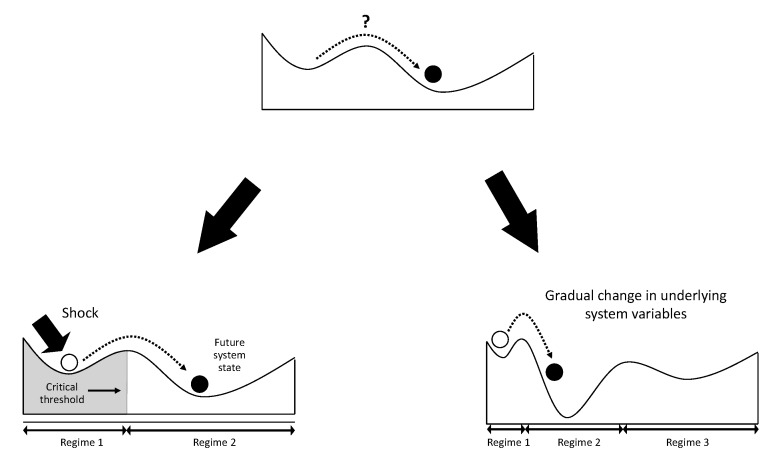
Regime shifts often result from a combination of slow ongoing gradual changes and an external shock to the system that tips the system into an alternative state (Adapted from [19]).

Regime shifts often result from a combination of slow ongoing gradual changes and an external shock to the system (Figure 1). Once the system is close to the threshold, a regime shift may be triggered by even a small shock to the system, such as a thunderstorm or protest action, that usually would not have any dramatic impacts. For instance, in the case of the food system, gradual changes in livelihood opportunities combined with climate shocks could trigger unexpected larger-scale changes in regional economies and food systems. Social–ecological regime shifts can have major impacts on human economies, security, and health, as they impact the supply of ecosystem services such as crop production and flood regulation [39,40,41]. Ecosystem services are the benefits that humans gain from the natural environment, which include, for instance, the production of water (provisioning services), the control of climate and disease (regulating services), nitrogen cycles and oxygen production (supporting services), and spiritual and recreational benefits (cultural services) [39]. Changes in ecosystem services directly impact on human wellbeing, including nutrition, livelihoods, social relations and freedom of choice.

The long-term sustainability of the system therefore relies on a deeper understanding of the mechanisms and drivers that lead to regime shifts, and the capacity to deal with such shifts when they occur. Resilience has been defined as the capacity of a system, subject to ongoing change, to continually self-organize and adapt in a way that retains the same function and structure, i.e., to withstand a regime shift [42]. However, social–ecological resilience is increasingly defined in a more normative way as the capacity of a social–ecological system to sustain human well-being, by adapting or even transforming in the face of change [43,44]. By this definition, regime shifts are seen as being conceptually similar to structural transformations that aim to create more desirable futures. In this conceptualization, the transformation to a sustainable future can be understood as a type of regime shift. Social–ecological regime shift theory highlights the notion that achieving such a transformation involves identifying and changing the dominant feedbacks that currently structure the system dynamics [45,46]. Innovations and innovators can be understood as key actors that help precipitate regime shifts/transformations by changing key systemic feedbacks in ways that change the depth of the basin or create new basins [47,48,49].

Although the idea of regime shifts as system transformations has started to recognize broader socio-political and economic contexts, these are not yet well integrated in social–ecological regime shifts theory [50]. A combination of perspectives from global food regimes and political–economy allows for a more complete diagnosis of the fundamental power structures and institutional landscapes that maintain systems on particular pathways. Social–ecological systems thinking, on the other hand, allows for an understanding of the complexities of systems, including the ways in which abrupt changes can be precipitated by interacting drivers and gradual shifts that reach a threshold or tipping point. Combining these perspectives offers potential insights for how to shift the global food system onto a new trajectory [47,51]. More generally, it is increasingly acknowledged that any sustainability transformation requires recognition of power and politics, as well as a systemic understanding of how complex social–ecological systems work and change [50,52,53]. In this regard, social and technical innovations can be particularly powerful in disrupting dominant system processes and creating the possibility for larger systemic transformations.

## 3. The role of Innovation in Regime Shifts

One of the critical areas for food system transformation pivots on behavior change and the aspirations and taste preferences of consumers of food, both in the Global North and South [10]. Current food consumption trends in the developed world—and increasingly in emerging economies—are unsustainable [54]. As people become more affluent, price indicators alone are unlikely to incentivize people to eat more sustainably or healthily. On the food consumption side, companies have invested heavily in shaping the food environment in which consumers choose food, through promoting foods that are aspirational and by creating novel foods that meet demands for tasty, convenient meals that often run counter to cultural traditions around food [55]. The high sugar and fat contents of these foods have been shown to have severe negative health consequences, especially for poorer households that cannot afford healthier alternatives or do not have physical access to fresh fruit and vegetables [56,57,58].

The food system provides an excellent case study for how innovation has gradually become supply driven rather than demand-driven. Demand-driven innovation is here characterized by companies creating interesting new ideas or products and then creating demand for that good by investing in practices such as excessive marketing: “need” for these goods is therefore manufactured [47]. In addition to those innovations that are predominantly profit-driven, there are other innovations, such as those arising from the public sector, that have also had unforeseen consequences on the food system. The innovations of the Green Revolution emphasized strengthening agricultural production by increasing crop yields through improved inputs: from seeds to machinery and irrigation [59,60]. Although these innovations have undoubtedly improved food security in terms of increasing yields and the total number of calories grown globally, they have ignored other dimensions of the food value chain, particularly concerning access and nutrition. The history of food system regimes presented in Section 4 offers three examples of how innovation has overcome certain challenges, whilst often creating larger problems; a consequence of innovation in a complex social–ecological system. The innovation of social–ecological systems, on the other hand, differs from these conventional innovation strategies because it is founded on notions of complexity, ambiguity, and diversity [47,61]. Examples of these will be the focus of Section 5.

### Transformative Social–Ecological Innovation

**Figure 2:**
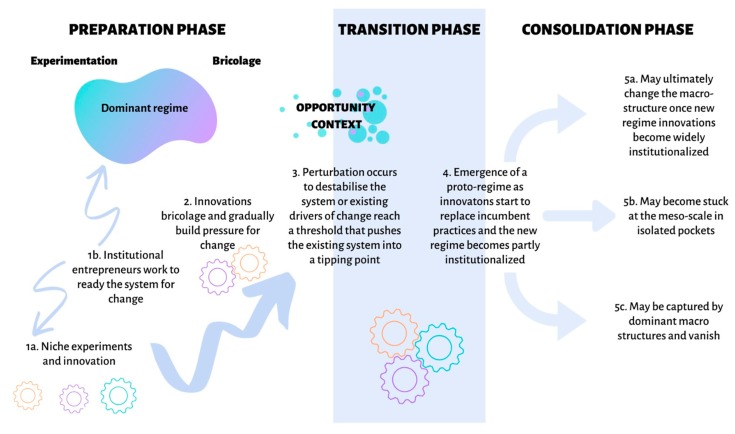
Three phases whereby innovations can cause a system to undergo a regime shift, or transform from one system state to another. The preparation phase requires innovations and experiments to be developed and over time to bricolage, whilst institutional entrepreneurs ready the system for change by bridging between the niche and the regime. A window of opportunity opens up when a perturbation weakens the incumbent system and this allows the innovations that have previously been marginal to become partly institutionalized during the transition phase. The third phase involves three pathways; a- where the resilience of the new regime is built and it becomes the new status quo; b- where the proto-regime becomes stuck and is isolated in pockets; c- where the dominant regime is so resilient that it adapts to the shock and reconfigures itself by capturing the innovations so that they do not result in transformative change. (Adapted from [65]).

Disruptive innovations can trigger transitions or transformation out of dominant regimes or stability landscapes into alternate regimes [47]. In social–ecological innovation, this tends to occur as a bottom-up rather than a top-down process, and thus, sits in contrast to corporate power that tends to be top-down [46,62]. At the local level, small, fast variables allow for rapid experimentation to occur; this can be equated with the ‘niche’ or safe space for innovation [63,64]. Most innovation does not survive, and so there is need for a continual process of design, redesign, and collapse, but every so often, when there is a conducive landscape, an innovation will be successful enough and intermediate processes, such as a more enabling governance arrangement, can start to formalize the novel innovation. At this stage, the innovation can become part of the dominant regime, or it can fail and return to the smaller system where social memory will allow for it to be adapted to the changing landscape environment (Figure 2).

There are three phases whereby niche innovations can cause a system to undergo a regime shift, or transform from one system state to another [53]. The preparation phase requires innovations at various levels of the system, often with institutional entrepreneurs operating simultaneously to weaken existing structures and create the possibility for change [53,66]. When a window of opportunity opens up, innovations that have been relatively marginal can suddenly be taken up and shape a new set of system processes, and a new regime. During the transition from one regime to another, small niche innovations can start to have impact at higher levels (See Figure 2). The third phase involves building the resilience of the new regime so that it is not vulnerable to returning either to its previous state or to a less desirable system state. This type of systemic transformation is not a managed process, whereby the final outcome is determined from the outset, but is an emergent one [53]. The system can be nudged (rather than managed) onto a particular trajectory, but the emergence of a new regime is a complex and unpredictable process.

In the next section, we describe three food system regime shifts, linked to three disruptive innovations. Such innovations play an important role in precipitating regime shifts, especially in guiding the direction that these shifts will take, although they in no way act in isolation, and the three we have chosen are examples that serve to illustrate the argument rather than definitive. By understanding the role of disruptive innovation in global food regime shifts, it is possible to speculate on how to nudge future innovations towards a more just and sustainable basin of attraction.

## 4. Three Examples of Food System Innovations Linked to Regime Shifts

The role that private sector actors play in shaping the global food system has become significant over the last century, especially as large corporations have consolidated the processes across the food system [23,67,68]. The array of actors involved in the food system and their changing roles are fundamental for understanding the regime shifts that the system has undergone over the last two centuries, largely through innovation in response to changing social and institutional contexts. Recognizing that innovations never act in isolation within systems, the following case studies illustrate how combining a political–economy and a social–ecological perspective of understanding regime shifts can help us comprehend some of the complex processes and interactions that, in retrospect, we can identify as regime shifts. By understanding the socio-political, ecological and economic contexts and drivers within which these important innovations occurred, the dimensions of the resulting regime shifts become clearer (See Table 1). These are by no means the only innovations in the food system, but by focusing on one critical intervention in the system, it is easier to conceptualize how change can be triggered, and therefore, how to intervene in a system to nudge it onto a more sustainable pathway. The case studies focus on innovations that occurred in the Global North that have had global impacts on the food system, stressing again where much of the power to shape the global food system has resided. Biophysical as well as social feedbacks necessitated adjustments in the capital allocation of powerful structures. This changing landscape allowed for other innovations to take hold to shift the food system into a new configuration of nature, people, and money.

### 4.1. Regime Shift 1: From Labor-Intensive Subsistence Agriculture to Commercial-Industrial Agriculture that could Feed Growing Cities: the Haber-Bosch Process to Produce Fertilisers and Subsequent Intensification Technologies

In the early nineteenth century, Europe was still largely an agrarian society, where the majority of the population cultivated food for a living. By the end of that century, as the industrial revolution took hold, peasants left the countryside for the cities and an industrial working class was formed [69]. As people moved from farms into urban areas, there was a need for a steady supply of produce from farms to feed the increasing number of workers in expanding cities. There was, however, less labor available on farms. The industrial revolution not only caused this problem, but also provided the means to a solution: mechanized agriculture. This mechanization coincided with the colonial-diasporic food regime that predicated a need for supplying food to European cities and workers from the colonies [22]. The industrial revolution resulted in increased mechanization of agriculture and a move away from it being labor intensive to being more reliant on other input costs, associated with the scientific discoveries of the age. The ‘disruptive innovations’ of the Industrial Revolution enabled Western societies to transcend a Malthusian assumption where it was believed that population growth would eventually outstrip natural resources, largely through revolutionizing the instruments of production and the division of labor [70].

A key disruptive innovation in this process was the Haber-Bosch process (of fixing N_2_ into NH_4_), first observed in 1909, that, after an over-capacity of the manufacture of explosives following World War 2, led to a refocus of the technology into the production of synthetic fertilizers [71]. Fertilizer in turn contributed to vast increases in the amount of food that could be produced on the same amount of land through intensification [72]. As more food was needed to supply burgeoning cities, this intensification of agriculture that increased yields from finite areas of farm land and animal bodies was a welcome development [36]. However, the new technological package of fertilizers, pesticides, heavy machinery, and new stock and crop varieties came at a significant environmental cost, with increasing reliance on industrial inputs, a reduction in internal natural cycles on farms, pressure on landscapes and animals, and other environmental impacts [36]. The combination of these interacting factors led to a reorganization of the system so that it functioned in a very different way, and can be thought of as a regime shift.

This change in the structure and function of the food system also impacted on the availability of livelihood and economic activities for people who had been agricultural laborers, as well as the quality of the food and nutritional content, impacting on human wellbeing. The outcomes of the Green Revolution were used to extend this regime to the Global South. Further, this trend created the space for companies to start specializing in agricultural inputs like farm machines, seeds, fertilizer, and pesticides, and for these to subsequently merge into the agribusiness giants of today, such as Monsanto (an agrochemical company that became a powerful seed company and has now been absorbed by Bayer, which describes itself as a ‘multinational pharmaceutical and life sciences company), Agrium (primarily a fertilizer company that is now called Nutrien after a merger with Potash Corp) [21,23], and Bunge, Cargill, Archer-Daniels-Midland, and Louis Dreyfud (the four largest grain trading companies that control up to 90% of the world’s cereal trade) [73]. Although cities throughout history have relied on food coming in from rural areas, i.e., the core has always been fed by the periphery, the scale on which industrialization enabled this relationship resulted in a growing disjuncture between food production and its consumption. The “distanciation” of the food system, as argued by Friedmann [74], was reinforced by the second major regime shift, which focuses on the power of global supermarket chains.

### 4.2. Regime Shift 2: From Local Traders to the Convenience of Global Supply Chains: the Establishment of Supermarkets and Fast Food Restaurants as Conventional Sites of Food Procurement

In response to people moving into cities and needing a central location for their food, the birth of the supermarket can be seen as a disruptive innovation in the food system, whereby one actor was responsible for supplying all the goods that would previously have been supplied by different actors (e.g., the goods from grocers, bakers, butchers etc. were now found under the same roof). The establishment of grocery stores was pioneered in Europe, which grew to dominate the global food retailing business a century later, providing an outlet for the food industry to sell its processed food products in the twentieth century. The first Sainsbury’s opened in London in 1869, where it grew into a privately listed company in 1922. The next biggest extant supermarket was founded by Albert Heijn in the Netherlands in 1887, becoming the company Ahold. The French followed with the establishment of the first Casino store in 1898. These retail giants were to become part of the food retail oligopoly that came to dominate the western world over the course of the twentieth century, spreading to all reaches of the globe through the connections established by the colonial system [75,76,77]. The remainder of this powerful group of food retailers was established in the mid-twentieth century; the United Kingdom’s Tesco’s in 1952, France’s Carrefour in 1958, and the USA’s Wal-Mart in 1962.

This second regime shift has at its core a new center of power within the global food system; the global supermarket chain that has profoundly impacted what a large proportion of the world eats, as well as how that food is produced, processed, and marketed [78]. According to Shaw, supermarkets were transformative for four main reasons [79]. Firstly, their patterns of innovation created Schumpeterian ‘disruptive competition’ (See [80]). More vibrant innovation was created because participants had to compete to offer more varied produce and service. Secondly, by continuously reinventing their format, processes, and channels of distribution, they created novel business models that became impossible to outcompete, as they were constantly adapting [79]. Thirdly, they transformed consumer culture in that customers came to expect a variety of products on offer in one convenient location where they were free to roam the aisles at their own pace [79]. Finally, their competitive edge and adaptive business model made them so successful that it led to the rapid growth of supermarkets, and so their influence spread quickly [79].

This model had universal applicability, and so, by the end of the 20th century, was transferred to a range of economies in a rapid expansion [81]. Having spread the business model throughout the USA and Western Europe, these companies became transnational, establishing themselves in foreign markets either directly or by getting their foothold in the market through partnerships with local companies [76,77].

A similar source of convenience in the food system arose on the other side of the Atlantic in the USA, and made its way across to Europe: the development of fast food restaurants. Schlosser’s book on the rise of the fast food chain in the United States gives a detailed account of the innovation that not only developed a model for getting cheap, convenient, and addictively tasty meals to all Americans, but also transformed how agricultural supply chains operated [82]. As the establishment of supermarkets allowed for the convenience of accessing all your food in one place rather than going to the fresh market, the baker, or the butcher, fast food chains provided ready-made food at a cheaper price than cooking it at home [83]. These innovative business models have continued to grow off each other in the twentieth century, and as technologies have provided increased means for efficiency, such as the microwave, the food industry has developed products accordingly, including ready-made meals high in salt, fat, and preservatives like sugar [57,84].

The development of these two models changed the rules of the game of the global food system and made different models of food access acceptable. Slow gradual changes pushed the food system, including both the agri-ecosystems upon which food production is based and the points where consumers access food, towards a threshold. Within a century, this enabled the food industry within the mercantile-industrial food regime to provide consumers with increasingly processed and unhealthy foods [56,57,84], directly impacting on human wellbeing and environmental integrity. The evolution of the agro-industrial complex in response to this second food regime is a defining feature of what constitutes the food that people access through these novel points of procurement.

### 4.3. Regime Shift 3: From Anonymous Global Supply Chains to Alternative Food Networks: the Rise of Corporate Responses to the Call for Increased Transparency

The phenomenon of the global expansion of supermarket chains into multinational corporations that established themselves in developing country markets since the 1990s is a product of the mercantile-industrial food regime [75,76,81]. These companies now have the network power to source and supply anything from everywhere; it has become commonplace to be able to find Indian mangoes, South African avocadoes, Kenyan green beans, and Chilean grapes in the same aisle in a single UK supermarket. However, the monopolization of the global food system by a few companies brought with it a backlash as consumers demanded transparency: they wanted to know more about what they were eating, how and by whom it was grown or reared or caught, where the seeds came from, and how many inputs were applied. The corporate response has been, by-and-large, the auditing of supply chains through standards from bodies like EUREP-GAP (now GLOBAL-GAP) [22]. This has been capital’s attempt to respond to consumer and activist demands about the anonymity of ‘Food from Nowhere’, i.e., the product of the globalized mercantile-industrial regime, to create ‘Food from Somewhere’ to sustain market share [85,86].

The rise of ‘alternative food networks’ is arguably a social movement critique of the increasing disconnect between the majority of consumers from the realities of the production of food. In other words, it reflects the transitioning from the mercantile-industrialist regime into what Friedmann (2005) posits as the corporate-environmental regime. As the environmental and social implications of the dominant food systems in the Global North have been recognized [10], alternatives have started to emerge, resulting in a potential means to rewire the system by altering the social–ecological feedbacks and dynamics [1]. These ‘alternatives’ include the organic, local, and Slow Food movements, but are increasingly also being mainstreamed by food labelling and certification from bodies like Fair Trade and the Forest Stewardship Council. As Goodman [87]: p. 10 notes:

This shift towards the production of quality local foods, as opposed to the generic ‘placeless’ commodities of productivist agriculture…is variously conceptualized as the re-embedding, resocializing, and re-localizing of food systems. Slow Food Supply Chains are a major institutional expression of these reconfigured production-consumption relations farmers are encouraged to ‘short-circuit’ industrial supply chains and to reconstruct the producer-consumer interface by engaging with different conventions and constructions of quality “that evoke locality/region or speciality and nature” (Marsden et al. 2000: p. 425).

Food businesses, as with other private sector entities that need to sell products, have become locked into an unsustainable, growth-oriented regime that has consumerism at its foundation. With a focus on optimizing shareholder value and externalizing social and ecological costs [47], it is unsurprising that innovation in this sector has only been quick to jump on the sustainability bandwagon when there are clear profits in sight (as per Friedmann’s third regime argument [22]). Innovations like ethical labelling, in particular, seek to create a sense of trust. ‘Fair Trade chocolate’, ‘Carbon Neutral wine’, ‘Rainforest Alliance coffee’, and ‘Organic cheese’ are certified labels that all impact production based on the preferences of ‘ethical’ consumers, and effectively places them and their certification bodies in charge of how food production is undertaken. This has interesting repercussions for farmers in the developing world, where many of the changes are taking place. Studies on the Roundtable on Sustainable Palm Oil have shown that despite an attempt at full equality and engagement among multiple stakeholders, the major processors and traders still dominate these discussions [88,89]. Fair Trade and other certification schemes similarly reflect good intentions in the North that do not necessarily translate into actual change, especially economically, for producers in the South [90,91]. Even the growth of ‘hipster’ culture and a reconnection to traditional ecological knowledge has instigated a cultural shift towards better quality, more ethical produce that can be seen in cities around the world, including the Global South [92,93,94]. This has, however, not yet been able to destabilize the current global food regime that continues to drive unsustainable and unhealthy practices [10]. Rather, it can be seen as an example of capture by the dominant regime of niche innovations that seek to address core social–ecological challenges (pathway 5c in Figure 2).

## 5. Quality, Taste, Cuisine, and the Role of Chefs as Social Innovators: Precursors for a Future Regime Shift

An idea or concept can transcend the stark binaries evident in food system debates—binaries that are often underpinned by ideological positions. The “farm to table” or “farm to fork” locavore movement that promotes serving local food at restaurants, preferably through direct acquisition from the producer, is an example of a mobilizing idea that has changed many people’s perceptions about where food comes from, reflecting some of the shifts in the third food regime described above ([96]). The idea of “farm to table” has, however, been limited, in that farmers end up serving the table, not vice versa, which makes good agriculture difficult to sustain. An example of a potential solution is Barber’s concept of cuisine, which encourages a collection of dishes that reflect a whole system of agriculture [97]. It comes from a similar perspective on food system transformation as espoused by Michael Pollan who emphasizes that change needs to come from a return to the skills of cooking and linking taste and nutrition directly to how food is produced and cooked [83,98].

Barber’s book “The Third Plate” doubles as a manifesto for the future of food and highlights the potentially transformative role that engaged chefs can make in the food system [97]. The manifesto argues for a radical shift in what a standard plate of dinner should look like, demonstrating that flavor must start with an understanding of soil, bringing emphasis to the provisioning and supporting of ecosystem services, and the role this plays in the quality of food (https://www.theguardian.com/lifeandstyle/2017/jan/15/dan-barber-mission-to-change-food-and-farming (Accessed 12 January 2020)). A central tenant is that a cook has a duty not only to know the farmers who provide his or her ingredients, but also to be actively involved from seed to table, by selecting for taste. There are increasing numbers of platforms around which chefs are starting to mobilize their role in healthy and sustainable food, including the Slow Food Chefs Alliance, which rallies around protecting biodiversity (https://www.fondazioneslowfood.com/en/what-we-do/slow-food-chefs-alliance/ (Accessed 12 January 2020)) and the Sustainable Development Goal 2 Hub’s Chef’s Manifesto on how the food industry can deliver a better food system ( http://www.sdg2advocacyhub.org/chefmanifesto (Accessed 12 January 2020)). Furthermore, the recognition of traditional cuisine and the knowledge of local cooks, not only chefs, especially in the Global South, offers further evidence of this movement gaining traction [99]. Another clear link can be made to SDG 12 on sustainable consumption and production. SDG 12 reflects the need to develop sustainability practices that bridge production and consumption; however, it is also the SDG most associated with trade-offs against achieving the other goals [100]. Strategic linkages between achieving the targets of SDG 12 through a food system lens could enable key leverage points to be identified that create synergies between the other SDGs, such as life below water (14) and life on land (15), rather than trade-offs.

Critiques of the notion of chefs supporting farmers need to be acknowledged, however. A prominent critic of the small farmer ideal is Julie Guthman, who has argued that agrarianism—which Barber and Pollan espouse—romanticizes the small-scale family farm (which can be an extraordinarily difficult lifestyle), reinforces patriarchy, and perpetuates injustice for farm workers [101]. These important questions bring to bear the issue of food justice and further emphasize why it is important to be able to analyze what is elevating these niches into potential new regimes, and what the pitfalls might be. Drawing these into the argument for strengthening the relationship between food, people, and planet, we can help identify and address many of the social and environmental issues that plague contemporary food systems.

A key question is how enough energy can be brought to a revolution and lead to a regime shift that is premised on sustainable and ethical practices in local contexts. This alternative regime holds great potential, not only from an environmental perspective, but by substantially improving human well-being by enhancing the quality of food and nutrition [93]. Countries in the Global South are often envisaged as passive recipients of regime-defining processes with little to no agency to act in the face of broader power structures. Strategies that build on and enhance local capacities are needed in these contexts rather than a new set of paternalistic solutions that do not address the complexity of the challenge. Emerging economies such as South Africa are confronted by the existence of multiple regimes coexisting within one country: a dual agrarian system consisting of a minority of white, commercial farmers who produce much of the country’s produce and a majority of small-scale black farmers [102], the triple burden of malnutrition (hunger, obesity, and micronutrient deficiencies), a high prevalence of diet-related diseases [103,104,105,106,107], and a diverse production landscape, vulnerable to climate change, with only 13% of land considered arable [108]. The South-African context, therefore, brings in a whole array of social–ecological and political economic issues linked to the food system, such as income inequality, malnourishment, obesity, food insecurity, water shortage, and soil degradation, amongst others.

Being able to tackle this complex array of interconnected concerns within the food system requires transformative change towards a more sustainable and just regime. It requires not only understanding the social–ecological implications, but also the political economic context, and how this needs to change. Unlike previous regimes that manifested as a result of the actions of powerful actors exploiting key innovations, we argue that the sustainability regime needs to disrupt from the bottom up, where local innovations and initiatives that meet local needs are able to effect systemic change whilst maintaining their contextual nuance. This theory of bottom up transformation follows the theory of transformative social–ecological transformation presented in Figure 2, and there are increasing examples of how this kind of change might be galvanized [109,110,111]. A key aspect of this approach lies in acknowledging diverse pathways of scaling impact: scaling up (getting bigger), scaling out (replicating), or scaling deep (changing norms and values) [99,112].

A study from Cape Town, South Africa has shown that niche eco-gastronomic initiatives like Bread Rev—a charity that teaches people to bake bread in wood-fired rocket ovens and sets up local community BREADshops—have the potential to create new interactions between humans and the environment [92]. For her MSc research, Markey (2017) conducted in-depth, semi-structured interviews with 13 niche actors in Cape Town’s bread, beer, gin, and pork industries to assess what potential these niche businesses had as seeds of transformation to disrupt and change the broader system [92]. Her findings showed that niche eco-gastronomic initiatives in the Greater Cape Town area have created new configurations of human-environmental relationships, whilst also addressing issues of inequality [92]. Despite being in different sectors, each of the actors was highly connected in response to the low institutional support for these small-scale businesses; however, exchange with the regime of large-scale producers remained limited [92]. Instead, niche actors mainly focused on scaling out and scaling deep to achieve change, fostering the further development of the eco-gastronomic sector, as well as engaging in conversations to shift perceptions and beliefs around what good food means and who can access it [92]. The businesses acted as brokers between producers and consumers to create new social–ecological relations through their eco-gastronomic initiatives, institutionalizing through their network a more holistic approach to creating social–ecological sustainability in their business models [92]. Although the approach to address these aspects varies depending on the specific local conditions and the business model, the study showed that the success of the innovations lay in the sharing of ideas and knowledge, experimentation, and creative usage of available resources, thus bricolaging new constellations of existing system components [92].

These examples are certainly not the only initiatives trying to drive food system transformation in South Africa. Innovations ranging from phone apps to help link small-scale fishers to consumers, organizations bridging indigenous knowledge holders, and marginalized producers with chefs and restaurants, and even social movements around access to land and agro-ecology, are all important in shaping a landscape from which a new food system can emerge [15,113,114,115,116]. At the core of these innovations is a disruption of the conventional, paternalistic solutions that are usually offered in response to food insecurity [34]. Rather than being externally-driven, these innovations have emerged from within their unique food environments to address the challenges that specific communities are facing and are looking at scaling in very different ways. Thus, these social–ecological innovations offer genuine alternatives to neoliberal strategies that tend to remove the agency from communities. By understanding how past innovations were captured and shaped the political–economy, it might be possible to guard against similar manifestations of power in the emergence of future regimes.

Similar innovations are underway in Europe, where a return to appreciating traditional ecological knowledge is gaining a following [94]. Links between traditional ecological knowledge and resilience in European landscapes of food production have been identified and called biocultural refugia [117,118]. This work focuses on how knowledge, experience, and practices of managing a local ecosystem and its services are captured, stored, revived, and transmitted through time as social memory [119]. The authors find that the biodiversity of many cultural landscapes has been maintained through local management practices, developed in the context of the relation between local environmental fluctuations and agricultural production. In Europe’s agricultural landscapes, loss of traditional ecological knowledge and practices has resulted in an associated erosion of biodiversity and regulating ecosystem services, leading to the conclusion that nurturing biocultural diversity is a fundamental principle for planetary stewardship [120]. Again, this shift acknowledges the agency of local knowledge-holders, thereby disrupting conventional top-down solutions that can be captured by powerful actors. Leveraging traditional knowledges and relationalities could allow for the emergence of an alternative global food system that is embedded in local realities, rather than in the powerful dominance of global actors who, during colonization and globalization, have been able to capture previous innovations to further their own ends.

Although social–ecological innovations currently remain niche, global shifts in discourses emphasizing more sustainable food systems could be reconfiguring the political economic landscape and opening up a window of opportunity for these to scale. By scaling appropriately, these alternatives could alter the feedbacks that maintain the status quo and allow for the global food regime to shift into an alternative state. There are significant numbers of examples of alternative, social–ecological innovations that are emerging in niches around the world [120]. Fostering an environment within which they may be able to flourish and influence the trajectory of the food system is becoming a key concern in creating a more sustainable global food system.

## 6. Concluding Perspectives

In this paper, we have argued that there is a novel academic contribution to be made by bringing together a political–economy conceptualization of food regimes with a social–ecological systems perspective of regime shifts in order to understand the complex dynamics of past transformations in the food system. We propose that this can best be done by unpacking three historical innovations (and their associated actors and power dynamics) that we believe have helped to enable past regimes shifts. These are meant to serve only as examples of how this approach can be used to understand better how food system regimes have shifted in the past, and are not meant to be seen as definitive. We argue that using a food regimes lens to learn how these innovations helped to create new food system configurations provides us with information on how to navigate future regimes that could address some of the key equity and sustainability challenges of the current global food system.

Analysis of past regime shifts highlights how the concept can be used to identify the main drivers responsible for shifting the system into an alternative state, and the feedbacks and power structures that maintain existing regimes. Reflecting on the nascent potential of future regime shifts, this can help identify leverage points or places to either push a failing system into tipping or intervening to facilitate transformation of the system [94]. However, we are cognizant that using such historical examples to learn about the future requires a level of reflexivity and humility in the claims that are made. Such an approach can benefit from insights from the field of historiography.

Mazlish [121] has argued for a similar conceptualization of measuring change in the past to that of regime shifts, namely by contributing the idea of a ‘historical rupture’ to mark abrupt change within an otherwise nuanced understanding of history, which describes change as being both continuous and discontinuous. As he contends, the lens through which understanding is gleaned, and in turn, a narrative produced and assertions made, is largely constructed by way of individual perspective, privilege, and purpose:

“Stringing together facts, in what often appears to be a functionally deterministic way, historians draw a smooth line through the past. Both the facts and the continuity are, we now realize, constructed. We construct what is a fact and recognize that we could have emphasized other ones. Equally important, however, is the recognition of ruptures in history… Against the view of the human past as marked by continuity, ruptures mark abrupt change.” (Mazlish 2011:p. 32).

In order to better understand food system regimes and regime shifts within these systems, it is crucial to understand the narratives of past changes, the actors who constructed them, and the agents and factors with the greatest relative impact on facilitating change. With a deconstruction of power and privilege behind past ruptures or shifts, today’s actors are better equipped to situate themselves within on-going and future regime shifts in order to bring about more desirable outcomes. With an awareness that the facts emphasized and narratives told are in great part happenstance constructs, a space opens up for the empowerment of actors to take an active role in not only reimagining past shifts, but in shaping new futures.

## Figures and Tables

**Table 1:** Summary of the regime shifts in the food system linked to key characteristics used to define these shifts.

	Regime Shift 1: Low-Input Labor-Intensive Farming to Commercial-Industrial Agriculture	Regime Shift 2: Food Procured from a Variety of Local Traders to Food Procured from a Supermarket	Regime Shift 3: From Anonymous Global Supply Chains to Alternative Food Networks
**Key innovations**	Haber-Bosch Process; Green Revolution technologies	Supermarkets; Fast and convenience foods	Certification and labelling; local and Slow Food movements
**Key drivers**	Industrial revolution Over-capacity in the manufacture of explosives after World War II Urbanization	Globalization of supply chains; Efficiency of international transport of goods	Consumer demands for transparency in value chain; Rise of ‘alternative food networks’
**Key feedbacks**	Increased agricultural production efficiency; Monoculture farming; Increased dependence on the companies providing inputs	Global expansion of supermarket chains; Disruptive competition; customer demand for variety of products; Cheaper food; Less time and skill spent on cooking	Higher percentage of income being spent on higher quality food; Growth in certification bodies and institutionalized auditing of supply chains; Greater costs to farmers to be enrolled; Support for local and niche food producers to enable their viability
**Key ecological impacts**	Increased pressure on land and animal bodies; Decreased agro-biodiversity; Decrease in water and soil quality; Increased emission of Greenhouse gases (GHGs); Loss of pollinators; Eutrophication of lakes and seas from agricultural run-off	Agricultural expansion leading to deforestation in the tropics; Increase in carbon emissions from transporting food around the world ‘food miles’; Increased meat production on feedlots emitting more GHGs and driving expansion of feed crops like soybean; Increased food waste; Reduced post-harvest losses as food is processed	Organic and less input intensive agriculture decreases impact on soils and water; Reduced food waste as food is more expensive; Improved agro-biodiversity
**Key social and health impacts**	Increase in calories available per person; Diversification of livelihood options away from agriculture; Shift from subsistence to commercial agriculture	Decrease in health due to increasingly processed and unhealthy foods being easily accessible and affordable; Increase in the variety of foods available; Exploitation of labor and land to meet international demand; Consolidation of food businesses into multinational corporations MNCs; Processing of food enables women to work and spend less time in the kitchen	Increased inequality in who can access good, healthy food; Improved conditions for those producers who can afford certification; Culinary knowledge valued; Improved nutrition for those who can afford better quality food
**Key references**	[70,71,72,95]	[57,75,76,78,79,81,82]	[87,88,89,90,91,92,94]
